# Association between *MYH9* and *APOL1* Gene Polymorphisms and the Risk of Diabetic Kidney Disease in Patients with Type 2 Diabetes in a Chinese Han Population

**DOI:** 10.1155/2018/5068578

**Published:** 2018-05-09

**Authors:** Hailing Zhao, Liang Ma, Meihua Yan, Yan Wang, Tingting Zhao, Haojun Zhang, Peng Liu, Yanzhen Liu, Ping Li

**Affiliations:** ^1^Beijing Key Lab for Immune-Mediated Inflammatory Diseases, Institute of Clinical Medical Science, China-Japan Friendship Hospital, Beijing, China; ^2^Clinical Laboratory, China-Japan Friendship Hospital, Beijing, China; ^3^Graduate School of Peking Union Medical College, Chinese Academy of Medical Science and Peking Union Medical College, Beijing, China

## Abstract

Single-nucleotide polymorphisms (SNPs) in *MYH9-APOL1* gene regions have been reported to be associated with diabetic kidney disease (DKD) in the American population. We examined the association between polymorphisms in *MYH9-APOL1* and DKD susceptibility in a Chinese Han population. *MYH9* rs3752462 (T>C) and *APOL1* rs136161 (C>G) were genotyped in 303 DKD patients and 364 type 2 diabetes mellitus (T2DM) patients without kidney disease using the TaqMan SNP genotyping assay. Chi-squared test and multivariate logistic regression were used to evaluate the association. We observed that only *MYH9* rs3752462 was associated with DKD (genotype, *P* = 0.004; allele, *P* = 0.002). Genetic model analysis revealed that rs3752462 was associated with increased risk of DKD under a dominant model adjusted by age and sex (adjusted odds ratio (aOR), 1.675; 95% CI 1.225–2.289; *P* = 0.001) and an additive model (TC versus TT: aOR, 1.649; 95% CI 1.187–2.290; CC versus TT: aOR, 1.817; 95% CI 0.980–3.367; *P* = 0.005). The combined effect of rs3752462 TC + rs136161 CC genotype showed an association of DKD adjusted by age and sex (aOR, 1.732; 95% CI 1.128–2.660; *P* = 0.012). After a Holm-Bonferroni correction for multiple tests, the C allele frequencies of the rs3752462 and the TC + CC genotype in the dominant model were considered statistically significant with a markedly increased risk of DKD (*P* < 0.00208; *P* < 0.002). Our results suggest that *MYH9* rs3752462 is significantly associated with an increased risk of DKD in Chinese Han individuals.

## 1. Introduction

Diabetic kidney disease (DKD) is a common and serious microvascular complication of diabetes mellitus (DM), which is characterized by an elevated urinary albumin excretion rate, elevated blood pressure, and declined renal function. Approximately 30–40% of DM patients will develop DKD, which is the leading cause of end-stage renal disease (ESRD) and renal failure [1]. Genetic factors appear critical in its pathogenesis based upon the evidence including aggregation in families, variable incidence rates of DKD between different races, and the highly heritable nature of diabetic renal clinic and histologic changes [2]. Compared to the Caucasian population, the Asia populations are more likely to suffer from DKD [3]. Identification of potentially susceptible genes and loci is needed to facilitate earlier identification and prevention of DKD, particularly in China.

The *MYH9* gene located on chromosome 22 q12.3-13.2 encodes nonmuscle myosin IIA. The approximately 224 kDa protein is widely expressed in most cells in the body [4, 5]. Polymorphisms in *MYH9* have been strongly associated with ESRD according to genome-wide association studies (GWAS), including human immunodeficiency virus- (HIV-) associated nephropathy in African Americans and idiopathic focal segmental glomerulosclerosis in European Americans and Hispanic Americans. Multiple common single-nucleotide polymorphisms (SNPs) in *MYH9* are associated with a greater risk for nondiabetic ESRD [6–8]. The *APOL1* gene, which is also located on chromosome 22, encodes apolipoprotein L-1. This gene has been associated with kidney disease in African Americans [9]. *APOL1* gene polymorphisms have also been more intensely associated with the risk of kidney disease previously attributed to *MYH9* [10]. The two genes cosegregate in many populations, which makes it difficult to differentiate between the two association signals.

A few studies reported the association of *MYH9* or *APOL1* with DKD. A GWAS and transethnic meta-analysis established the significant associations of *MYH9* and *APOL1* on chromosome 22 q12.3 with DKD in European American, African American, and American Indian populations. Furthermore, *MYH9* (rs5750250)-*APOL1* (rs136161) contributed the strongest association with DKD in African American populations [11]. Another study reported the association of four *MYH9* SNPs (rs4821480, rs2032487, rs4281481, and rs3752462) with T2DM-ESRD susceptibility in European Americans [8]. However, among them, only *MYH9* rs3752462 and *APOL1* rs136161 present genetic polymorphisms in Chinese Han individuals.

The susceptibility of *MYH9* and *APOL1* polymorphisms with DKD in Chinese populations has not been well studied. Considering the strong ethnic heterogeneity for gene polymorphisms, we evaluated the association of *MYH9* rs3752462 (T>C) and *APOL1* rs136161 (C>G) with DKD in a Chinese Han population.

## 2. Materials and Methods

### 2.1. Subjects

The clinic-based, case-control study recruited a total of 667 volunteers with T2DM. Among them, 303 patients with a history of DKD were defined as the case group. The remaining 364 participants, who had been diagnosed as T2DM for at least 7 years and had no history of DKD, were defined as the control group, regardless of age and sex.

Type 2 diabetes patients were diagnosed according to the 2012 American Diabetes Association diagnostic criteria; DKD patients were defined by the National Kidney Foundation Kidney Disease Outcomes Quality Initiative (NKF K-DOQI) guidelines.

This study was approved by the institutional ethics committee of the China-Japan Friendship Hospital (Beijing, China), and written informed consent was obtained from all individuals.

### 2.2. DNA Isolation and Genotyping

Genomic DNA was extracted from peripheral blood using the QIAamp DNA Blood Mini Kit (Qiagen, Hilden, Germany) in accordance with the manufacturer's protocol and quantified using a NanoDrop 1000 spectrophotometer (Thermo Scientific, Waltham, MA, USA). Genotyping was confirmed using the TaqMan SNP genotyping assay (Applied Biosystems, Waltham, MA, USA) and the ABI PRISM 7500 Sequence Detection System (Applied Biosystems).

Polymerase chain reaction (PCR) amplification was performed in a 25 *μ*L reaction mixture containing 50 ng DNA, 12.5 *μ*L of Premix Ex Taq (Takara, Shiga, Japan), 5 pmol of each primer (Applied Biosystems), and 3 pmol of each probe (Applied Biosystems). The amplification conditions consisted of 40 cycles of 95°C for 10 min, 92°C for 15 seconds, and 60°C for 1 min. The primers used to detect SNPs were synthesized by Applied Biosystems.

To verify genotypes, the PCR products were randomly selected for DNA sequencing analysis by TsingKe Biological Technology (Beijing, China) and the results were compared with the results of TaqMan genotyping. The primers used for the PCR were rs3752462, 5′-AAGACACCTCCACAACCAACAC-3′ (forward) and 5′-GCTCTTCAACCACACCATGTTC-3′ (reverse), and rs136161, 5′-CTCTCTTGCTG GCTTATGGAA-3′ (forward) and 5′-GCTGTGATGTGGGACTTGTTT-3′ (reverse).

### 2.3. Statistical Analyses

Clinical data, including age, gender, body mass index (BMI), blood pressure, duration of diabetes, A1C, total cholesterol (TC), high-density lipoprotein cholesterol (HDL-C), low-density lipoprotein cholesterol (LDL-C), triglyceride (TG), and homocysteine (Hcy), were non-Gaussian distributed. Wilcoxon signed-rank test was used to analyze the differences in clinical characteristics of the DKD and diabetic groups, and the data are presented as median (interquartile range). The Hardy-Weinberg equilibrium analysis of both SNPs was conducted using the Chi-squared test. The genotype and allelic frequencies of SNPs were also assessed by the Chi-squared test.

Multivariate logistic regression was carried out to analyze the association between each SNP and susceptibility to DKD after adjustment for age and gender in the additive, recessive, or dominant models. Multivariate logistic regression was also used for the combined effect of both *MYH9* rs3752462 and *APOL1* rs136161 polymorphism on DKD. An example to define these genetic models is the rs3752462 SNP, where C is the minor allele. For the dominant model, CC and TC were coded as 1 and TT was coded as 0. For the recessive model, CC was coded as 1 and TC and TT were coded as 0. For the additive model, CC, TC, and TT were coded as 2, 1, and 0, respectively. For SNP rs136161, G is the minor allele.

The Holm-Bonferroni correction was started by ordering the *P* values (from lowest to highest) as *P*_(1)_ … *P*_(*m*)_ and let the associated hypotheses be *H*_(1)_ … *H*_(*m*)_. For a given significance level *α*, let *κ* be the minimal index such that *P* > *α*/(*m* + 1 − *κ*). Reject the null hypotheses *H*_(1)_ … *H*_(*κ* − 1)_ and reject *H*_(*κ*)_ … *H*_(*m*)_. In present research, association analysis was performed for 26 times, *m* = 26.

## 3. Results

### 3.1. Subjects

A total of 667 T2DM participants were included, including 303 patients with a history of DKD and 364 patients without a history of kidney disease. The clinical characteristics of the participants are listed in Table 1. There were no significant differences in gender, smoking, TC, and LDL-C between the two groups. However, there were significant differences in age, BMI, duration of diabetes, blood pressure, A1C, Hcy, HDL-C, and TG between the DKD and T2DM groups.

### 3.2. Genotype and Allele Distributions of *MYH9* rs3752462 and *APOL1* rs136161 Polymorphisms

The genotype and allele frequencies of the rs3752462 and rs136161 polymorphisms in DKD and T2DM groups are shown in Table 2. Both SNPs were in Hardy-Weinberg equilibrium (*P* > 0.05), and their minor allele frequencies were >5%. The genotype and allele frequencies of the rs3752462 were different between DKD and T2DM groups (genotype, *P* = 0.004; allele, *P* = 0.002). A significant association with the increased risk of DKD remained for the minor allele C after a Holm-Bonferroni correction (*P* < 0.00208). However, there was no significant differences in the genotype and allele frequencies of the rs136161 between the two groups (genotype, *P* = 0.944; allele, *P* = 0.751).

### 3.3. Association of *MYH9* rs3752462 and *APOL1* rs136161 Polymorphisms with DKD

Different genetic models were applied to verify the associations of *MYH9* rs3752462 and *APOL1* rs136161 polymorphisms with DKD (Table 3). We assumed that the minor alleles of both SNPs were the risk factors compared to the common alleles. In the dominant model, multivariate logistic regression analysis revealed that when the rs3752462 TT genotype was used as the reference, the TC + CC genotype was associated with a high risk of DKD (TC + CC versus TT: odds ratio (OR), 1.690; 95% CI 1.240–2.303; *P* = 0.001). In the additive model, when the rs3752462 TT homozygote genotype was used as the reference, the TC and CC genotypes were associated with a decreased risk of DKD (TC versus TT: OR, 1.684; 95% CI 1.216–2.332; CC versus TT: OR, 1.720; 95% CI 0.935–3.162; *P* = 0.004). In the recessive model, when the rs3752462 TT + TC genotype was used as the reference, the CC genotype was not associated with the risk of DKD (CC versus TT + TC: OR, 1.398; 95% CI 0.772–2.533; *P* = 0.269). The results were remained similar after adjusted by age and sex. After the Holm-Bonferroni correction, only the TC + CC genotype showed significant association with an increased risk of DKD (*P* < 0.002). There was no significant association between *APOL1* rs136161 and the risk of DKD under these genetic models, which was remained similar after adjustment for age and sex (Table 3).

### 3.4. Combined Effect of *MYH9* rs3752462 and *APOL1* rs136161 Polymorphisms on DKD

Multivariate logistic regression analysis was applied to analyze the combined effect of both SNPs on DKD (Table 4). When the rs3752462 TT + rs136161 CC genotype was used as the reference, we found that the patients with rs3752462 TC + rs136161 CC genotype showed a higher risk of DKD (OR, 1.734; 95% CI 1.134–2.652; *P* = 0.011). The combined effect of other genotypes of rs3752462 and rs136161 was also examined; however, no difference was found. After adjustment by age and sex, the results were similar (Table 4). However, there was no significant association after Holm-Bonferroni correction.

## 4. Discussion

DKD is one of the most frequent microvascular complications of diabetes and is the leading cause of ESRD worldwide. Genetic heterogeneity and gene-gene or gene-environment interactions are frequently hypothesized as being important in this complex genetic disorder [12]. In the present study, 667 participants (303 DKD patients and 364 DM patients) were enrolled to investigate associations of *MYH9* rs3752462 and *APOL1* rs136161 with the risk of DKD in a Chinese Han population. Our results indicate that the minor allele of SNP rs3752462 is associated with an increased risk of DKD, while *APOL1* rs136161 was not significantly associated with DKD. The results suggest that *MYH9* rs3752462 might play an important role in the risk of DKD in the Chinese Han population.

The first association study of *MYH9* with kidney disease was observed in the patients with the giant platelet syndromes, a group of diseases caused by *MYH9* mutations and with a spectrum of abnormalities including low platelet count, hearing loss, giant platelets, and cataract may present focal segmental glomerular sclerosis (FSGS) [7]. *APOL1* is a neighbor gene presenting very strong cosegregation with *MYH9* in African descendants. Two studies reported stronger association with CKD of *APOL1* than *MYH9*, being the marker possibly responsible for the effect previously attributed to *MYH9* [9, 13]. Previous studies suggested that common polymorphisms in *MYH9* were strongly associated with nondiabetic kidney diseases in several ethnic populations [14–16]. However, polymorphisms in *MYH9* have been proven to be associated with diabetic nephropathy in 1963 European Americans, including 536 cases with T2DM-ESRD and 1427 nonnephropathy controls [8]. A recent GWAS and transethnic meta-analysis showed that SNPs in the *MYH9-APOL1* gene region showed the strongest association with DKD in African Americans, which provided suggestive evidence for association with DKD, although it did not reach genome-wide significance (*P* < 5 × 10^−8^) [11]. Our results indicated that *MYH9* rs3752462 was strongly associated with clinically diagnosed DKD in a Chinese Han population, and the minor allele C contributed to the increased risk of DKD, which might be due to genetic heterogeneity. Although the genotype and allele frequencies of *APOL1* rs136161 were not associated with DKD, SNP rs136161 CC genotype combined with SNP rs3752462 TC genotype showed association with the risk of DKD, providing further evidence that single genetic abnormalities were rarely the only cause of complex disease.

*MYH9* encodes the nonmuscle myosin heavy chain 9, which forms myosin II with other subunits. Myosin II, a motor protein, binds actin to regulate cellular motility. *MYH9* is mainly expressed in the podocytes, as well as in mesangial cells and arteriolar and peritubular capillaries in kidneys. As a motor protein, abnormal *MYH9* expression, localization, or function change will lead to cytoskeleton damage, further causing proteinuria or renal failure in patients with CKD [17–19]. Classical deletion of *Myh9* in mice results in embryonic lethality due to the loss of cell-cell adhesion and cell movement during gastrulation [20, 21]. Podocyte­specific deletion of *Myh9* in C57BL/6 mice causes significant susceptibility to experimental doxorubicin hydrochloride glomerulopathy [22]. A common missense mutation in MYH9 (E1841K) alters podocyte cytoskeletal structure and renders podocytes more susceptible to injury after a damaging stimulus [23]. However, the mechanisms responsible for them have not been defined. Wasik et al. found that the activity of myosin II was reduced by septin 7, which could hinder GLUT4 storage vesicle and fusion with the plasma membrane, reduce glucose uptake into podocytes, and further cause insulin resistance [24]. Fan et al. showed that NM-IIA activity was also inhibited by SLIT2/ROBO2 signaling, which could reduce podocyte adhesion in kidney glomeruli and aggravate the injuries of the glomerular filtration barrier in patients with CKD [25].

SNP rs3752462 is located in intron 13 of *MYH9*, and the functional effect has not been reported. One possibility was that the mutation in noncoding areas modulated gene transcription or pre-mRNA splicing. Furthermore, *MYH9* rs3752462 might directly regulate gene expression or on a more distant gene (such as *APOL1*). It has been reported that the strongest kidney disease association was mapped to the region of introns 13–15 by genotyping 79 *MYH9* SNPs in a total of 2496 cases (FSGS, HIV-associated nephropathy, and ESRD attributed to hypertension) and healthy controls [26]. Therefore, SNP rs3752462 in *MYH9* might be a functional variation or just a tag SNP in strong linkage disequilibrium with the causal functional SNP. This hypothesis needs confirmation in a future study.

Recently, *MYH9* rs3752462 was associated with cerebrovascular blood flow (CBF) in patients with type 2 diabetes [27]. Both cerebrovascular disease and DKD were the main vascular complications of T2DM, which implies similar molecular mechanisms in the association of *MYH9* rs3752462 with cerebrovascular disease and DKD. In addition, the T allele of SNP rs3752462 was associated with an excess risk for high blood pressure in patients with CKD in a Chinese population. It was also revealed that SNP rs3752462 was an independent predictor of a reduced glomerular filtration rate in the Spanish RENASTUR cohort population [28]. Therefore, blood pressure or glomerular filtration rate might be involved in the risk of *MYH9* rs3752462 on DKD.

The association of *MYH9-APOL1* is very interesting in DKD. It could provide the genetic tool to identify DM patients with increased risk of progressing to DKD, at least in populations in whom the mutations are known to be prevalent variations. Comprehension of the biological mechanisms determining proteinuria and CKD in patients presenting these mutations can create opportunity for new therapeutic targets and measures.

In conclusion, our study suggests that *MYH9* SNP rs3752462 is significantly associated with DKD in patients with DM and confirms the minor allele C as a risk factor of DKD. *APOL1* rs136161 was not related to the risk of DKD in our sample, but the result can be related to our small sample size. It will be a current challenge to explore the biological mechanisms underlying this association in future research.

## Figures and Tables

**Table 1:** Demographics and clinical characteristics of T2DM patients with and without kidney diseases.

Variables	DKD (*n* = 303)^a^	DM (*n* = 364)^a^	*P*
Age (y)	63.0 (55.0, 72.0)	61.0 (54.0, 68.0)	0.002
Sex, male (%)	63.4 (192/303)	58.0 (211/364)	0.156
BMI (kg/m^2^)	25.82 (24.0, 28.23)	25.29 (23.2, 27.78)	0.008
Duration of diabetes (y)	15.0 (9.0, 21.0)	13.0 (10.0, 18.0)	0.039
History of hypertension (%)	77.56 (235/303)	48.08 (175/364)	<0.001
Current smoking (%)	33.0 (100/303)	26.6 (97/364)	0.073
SBP (mmHg)	138.0 (125.0, 150.0)	126.0 (120.0, 139.8)	<0.001
DBP (mmHg)	80.0 (75.0, 84.0)	80.0 (70.0, 80.0)	0.026
A1C (%)	8.0 (6.7, 9.35)	7.5 (6.5, 9.2)	0.037
Hcy (*μ*mol/L)	13.89 (11.09, 17.22)	11.49 (9.54, 13.62)	<0.001
TC (mmol/L)	4.21 (3.49, 5.06)	4.17 (3.55, 4.92)	0.51
HDL-C (mmol/L)	0.96 (0.78, 1.18)	1.02 (0.85, 1.24)	0.004
LDL-C (mmol/L)	2.34 (1.86, 3.05)	2.38 (1.91, 2.96)	0.74
TG (mmol/L)	1.74 (1.22, 2.57)	1.43 (1.0, 2.17)	<0.001

Abbreviations: A1C, hemoglobin A1C; BMI, body mass index; DBP, diastolic blood pressure; Hcy, homocysteine; HDL-C, high-density lipoprotein cholesterol; LDL-C, low-density lipoprotein cholesterol; SBP, systolic blood pressure; TC, total cholesterol; TG, triglyceride. ^a^Data are shown as median (interquantile range) or %. *P* < 0.05 indicates statistical significance.

**Table 2:** Genotype and allele frequency of SNPs rs3752462 and rs136161 between DKD patients (*n* = 303) and DM controls (*n* = 364).

	Genotypes, *n* (%)					Alleles, *n* (%)		
rs3752462	TT	TC	CC	HWE *P* value	*P*	T	C	*P*
DM	227 (62.4%)	115 (31.6%)	22 (6.0%)	0.155	0.004^∗^	569 (57.1%)	159 (42.9%)	**0.002** ^∗^^,∆^
DKD	150 (49.5%)	128 (42.2%)	25 (8.3%)	0.752		428 (70.6%)	178 (29.4%)	
rs136161	CC	CG	GG	HWE *P* value	*P*	C	G	*P*
DM	213 (58.5%)	126 (34.6%)	25 (6.9%)	0.287	0.944	552 (75.8%)	176 (24.2%)	0.751
DKD	180 (59.4%)	104 (34.3%)	19 (6.3%)	0.449		464 (76.6%)	142 (23.4%)	

^∗^ *P* < 0.05; ∆ indicates statistical significance by Holm-Bonferroni correction; HWE: Hardy-Weinberg equilibrium.

**Table 3:** Genetic model analyses of the association between the SNPs and DKD with adjustment for age and gender.

	Genetic models	Genotypes	DKD	DM	Without adjustment		With adjustment	
					OR (95% CI)	*P*	OR (95% CI)	*P*
rs3752462	Additive	TT	150 (49.5%)	227 (62.4%)	1^#^	0.004^∗^	1^#^	0.005^∗^
		TC	128 (42.2%)	115 (31.6%)	1.684 (1.216–2.332)		1.649 (1.187–2.290)	
		CC	25 (8.3%)	22 (6.0%)	1.720 (0.935–3.162)		1.817 (0.980–3.367)	
	Dominant	TT	150 (49.5%)	227 (62.4%)	1^#^	0.001^∗^^,∆^	1^#^	0.001^∗^^,∆^
		TC + CC	153 (50.5%)	137 (37.6%)	1.690 (1.240–2.303)		1.675 (1.225–2.289)	
	Recessive	TT + TC	278 (91.8%)	342 (94.0%)	1^#^	0.269	1^#^	0.193
		CC	25 (8.2%)	22 (6.0%)	1.398 (0.772–2.533)		1.493 (0.817–2.728)	

rs136161	Additive	CC	180 (59.4%)	213 (58.5%)	1^#^	0.944	1^#^	0.901
		CG	104 (34.3%)	126 (34.6%)	0.977 (0.704–1.354)		1.011 (0.726–1.407)	
		GG	19 (6.3%)	25 (6.9%)	0.899 (0.480–1.686)		0.869 (0.460–1.644)	
	Dominant	CC	180 (59.4%)	213 (58.5%)	1^#^	0.816	1^#^	0.933
		CG + GG	123 (40.6%)	151 (41.5%)	0.964 (0.707–1.314)		0.987 (0.721–1.350)	
	Recessive	CC + CG	284 (93.7%)	339 (93.1%)	1^#^	0.757	1^#^	0.652
		GG	19 (6.3%)	25 (6.9%)	0.907 (0.489–1.681)		0.866 (0.463–1.618)	

Abbreviations: ORs, odds ratios; CI, confidence interval. ^#^Reference category (odds ratio, 1.0); ^∗^*P* value < 0.05; ∆ indicates statistical significance by Holm-Bonferroni correction.

**Table 4:** The combined effect of *MYH9* rs3752462 and *APOL1* rs136161 polymorphisms on DKD.

Genotypes		DKD	DM	Without adjustment		With adjustment^¶^	
rs3752462	rs136161	303	364	OR (95% CI)	*P*	OR (95% CI)	*P*
TT	CC	89	134	1^#^	—	1^#^	—
TT	CG + GG	61	93	0.988 (0.649–1.503)	0.953	1.039 (0.679–1.588)	0.861
TC	CC	76	66	1.734 (1.134–2.652)	0.011^∗^	1.732 (1.128–2.660)	0.012^∗^
TC	CG + GG	52	49	1.598 (0.995–2.565)	0.052	1.596 (0.989–2.575)	0.055
CC	CC	15	13	1.737 (0.789–3.826)	0.170	1.923 (0.864–4.283)	0.109
CC	CG + GG	10	9	1.673 (0.654–4.281)	0.283	1.736 (0.669–4.503)	0.257

^#^Reference category (odds ratio, 1.0); ^¶^Adjustment for age and gender; ^∗^*P* value < 0.05.
